# Policy-Relevant Indicators of Urban Emergency Medical Services COVID-19-Patient Encounters

**DOI:** 10.1007/s11524-022-00672-0

**Published:** 2022-11-02

**Authors:** Mark Brennan, Justin Steil, Sophia Dyer, Laura Segal, James Salvia, Erin Serino

**Affiliations:** 1 Boston Emergency Medical Services, 76 Albany Street, Boston, MA 02111 USA; 2 Massachusetts Institute of Technology, 77 Massachusetts Avenue, Cambridge, MA 02134 USA

## Abstract

In the first two years of the COVID-19 pandemic, members of Boston Emergency Medical Services, the City of Boston’s municipal ambulance service, had 7,689 encounters with confirmed-positive Boston residents. As COVID-19 virus strains continue to infect residents in Boston and across the country, understanding the correlation between population positivity, EMS encounters, and hospitalizations can inform healthcare response. This study examines urban virus-surveillance indicators that can serve as an early warning of the volume of Emergency Medical Services (EMS) encounters with COVID-19 positive patients and subsequently how EMS encounters with confirmed COVID-19 patients can serve as an early indicator of future hospital-demand surges. With daily data from Boston EMS and three other public agencies, we evaluate the relationship between five indicators and confirmed Boston EMS COVID-19 encounters by estimating separate Auto Regressive Integrated Moving Average models and cross-correlating their residuals. This study finds a significant and positive correlation between new COVID-19 cases citywide and EMS encounters 6 days later (p < 0.01), as well as between confirmed EMS encounters with COVID-19 patients and the number of intensive care unit beds occupied 7- and 18 -days later (p < 0.01). This study provides city health leadership needed clarity on the specific ordering and associated time lag in which infections in the population increase, EMS members encounter positive patients, and hospitals deliver care.

**Supplementary Information:**

The online version contains supplementary material available at 10.1007/s11524-022-00672-0.

## Introduction

To plan for the evolving waves of infection associated with a pandemic or even endemic virus response, city health leadership can be more strategic in their response with clarity on the specific ordering in which an outbreak spreads, ambulance services transport sick patients, and the hospital system delivers critical and sub-acute care. EMS serves as both an essential public safety service and early-warning mechanism in urban health systems. By identifying the intervals at which more COVID-19-positive patients needing emergency medical care (“EMS encounters”) intermediates rises in community infection and growing demand for hospital care, city health officials and partners can be more informed in planning and executing surges in medical-care capacity.

First, forecasting demand for emergency medical care enables responses that are effective for patients and safe for frontline personnel. EMS systems play a key role in urban health care delivery, disproportionately helping city residents with low-incomes overcome economic and spatial barriers to definitive care by providing emergency care and transportation to hospitals. Existing analyses show how weather and day of the week, among other factors, affect demand for emergency medical care [1–3]. Analysis relating indicators built from population virus surveillance to EMS encounters with COVID-19 positive patients, however, is limited.

Second, EMS caseloads can serve as an early-warning mechanism in urban healthcare systems [4, 5]. Trends in EMS COVID-19 encounters can reflect both community prevalence of the virus as well as virus morbidity. The pandemic has directly impacted hospital capacity, and early indications from EMS can, at the city-level, serve as a supplementry data point, informing hospital planning and operational decisions. Anticipating demand on the healthcare system from COVID-19 infections remains difficult [6]. While recent scholarship has found that EMS encounters with suspected COVID-19 patients correlate with new COVID-19 patient hospitalizations [7], there is still limited research on the correlation between confirmed encounters with COVID-19 patients, a consistent measure across jurisdictions, and indicators of healthcare system capacity.

We focus on Boston to help fill these two gaps in evidence in urban health care delivery. Boston was one of the first COVID-19 epicenters in the USA, making insights from it particularly applicable to planning for future pandemics in urban settings. As one of the few major urban EMS located within a public health department, Boston EMS sustained the ability to verify COVID-19 positive patient encounters, allowing for trend analysis from the onset of the pandemic. With 27 frontline ambulances and almost 400 EMS personnel, Boston EMS serves an estimated daily population of 1.2 million people and from March 2020 to November 2021 (21 months), responded to 198,682 incidents. In some weeks during that timeframe, as much as 15.8% of calls were confirmed to be COVID-19 positive.

## Methods

We use unique data from Boston EMS and three other local and state agencies to correlate trends in population and healthcare-system indicators with EMS encounters with confirmed COVID-19-positive EMS patients encounters (“EMS encounters”). We study daily data from March 1, 2020 to November 30, 2021, which encompasses three distinct waves of the COVID-19 pandemic in Boston. The data sources and full definitions of the variables are presented in Appendix Table A1.1.

The primary variable of interest is EMS encounters with patients confirmed positive for COVID-19 (“EMS encounters”), which, as a bureau of the Boston Public Health Commission, was possible to construct by vetting daily COVID-19 positive lists with EMS records to verify encounters.

We identify two data sources from virus surveillance that we hypothesize could help forecast future EMS encounters with COVID-19-positive patients: (1) the Massachusetts Water Resources Authority pilot study with Biobot Analytics, a wastewater epidemiology company, of SARS-CoV-2 genetic material in the wastewater arriving at the greater Boston wastewater treatment plant (“wastewater rate”); and (2) the daily number of new COVID-19 cases based on testing citywide reported to the state.

Next, we identify three data sources from virus surveillance and capacity tracking efforts in the Boston hospital system that we hypothesize recent EMS encounters with COVID-19-positive patients may predict: (1) the number of patients making ED visits related to COVID-19 at emergency departments city-wide (“ED COVID-19 patients”); (2) new hospitalizations of COVID-19-positive patients citywide (“hospitalized COVID-19 patients”); and (3) the number of intensive care unit (ICU) beds occupied in Boston hospitals (“ICU-occupied beds”).

Using the Box-Jenkins method to analyze these data, the study cross correlates the indicators with EMS encounters to reveal significant correlations and at what lags or leads. Each time series was rendered stationary using a first difference, and for EMS encounters and hospitalized COVID-19 patients the process variance was reduced with a log-based transformation. For new cases, 7-day variation was netted out (inherent to much COVID-19-related data) [8]. These standard adjustments are detailed in Appendix Table A1.2. We apply a root test to confirm stationarity [9]. For each time series, we fit an auto regressive integrated moving average (ARIMA) model and generate independent residuals that are not autocorrelated. We measure the model fit with Akaike Information Criterion and check for any autocorrelation within model residuals with the Box-Ljung test [10]. These models are presented in Appendix Table A1.3. Third, the residuals between EMS encounters and the indicators were cross correlated. This allows us to check if two time series are meaningfully correlated by measuring coincident short-term variation in the residuals of each series [11].

## Results

**Fig. 1:**
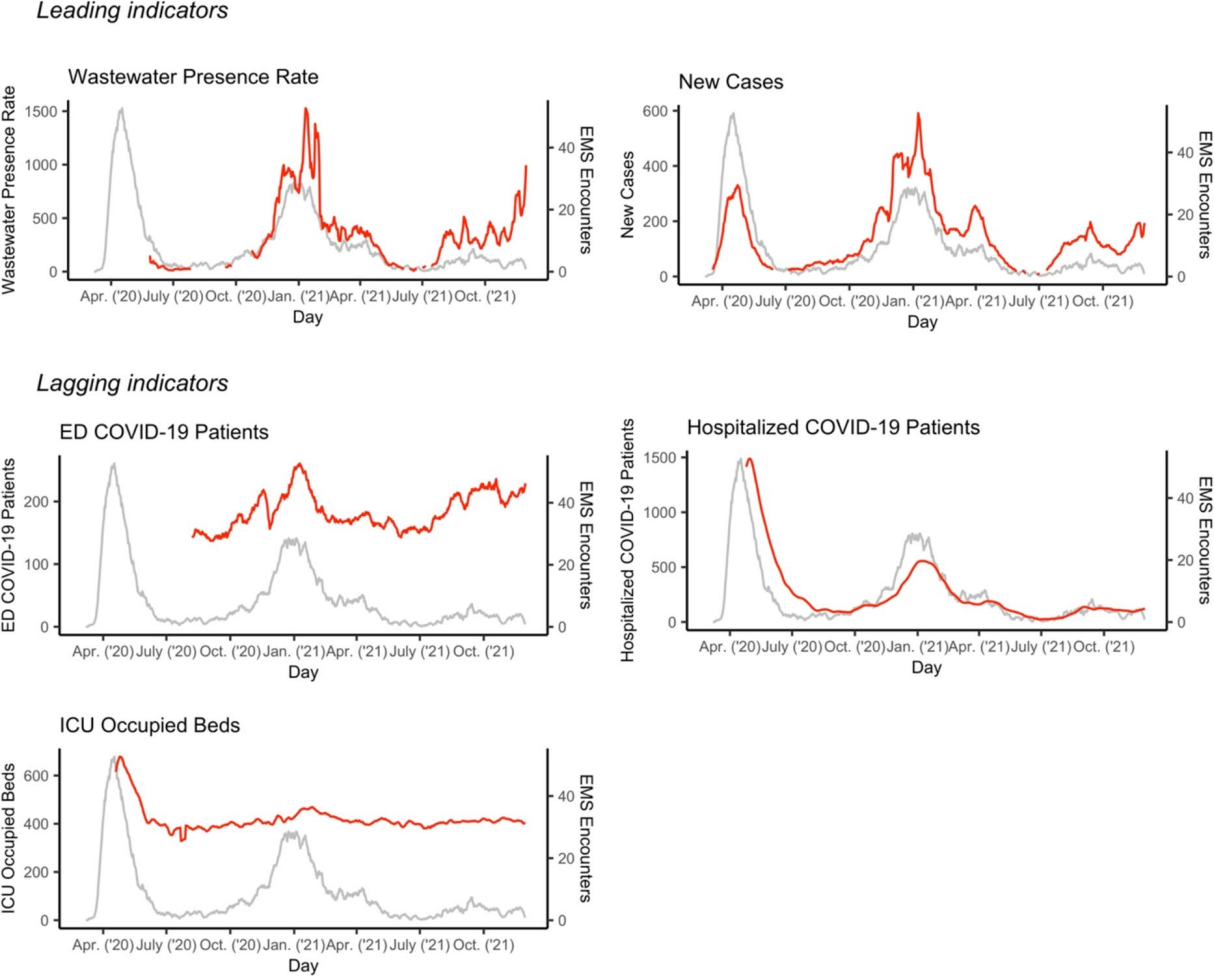
Trends in EMS COVID-19-patient encounters and leading and lagging indicators. Note*:* These panels present the trends for EMS COVID-19-patient encounters and indicators. The gray line (constant across all graphs) represents daily EMS encounters. The red line is the indicator

There is a strong degree of overall correlation in the trends between EMS encounters and most of the indicators, as seen in Fig. 1. The three distinct waves are apparent in EMS encounter and most indicator trends.

Appendix Figure A2.1 shows the cross-correlation coefficient of the residuals by daily lag (lead) with confidence intervals. Simple correlation coefficients between time series are reported in Appendix Figure A2.2. Among the leading indicators of EMS encounters, we observe a significant and positive correlation between the 6-day lead in new cases and EMS encounters (*p* < 0.01). The presence in wastewater does not correlate significantly with EMS encounters, though early variance and missingness in the spring of 2020 may obscure a relationship. Among the lagging indicators, we report significant and positive correlations between EMS encounters and ED COVID-19 patients at a 1-day lag (*p* < 0.01) and ICU occupied beds at 7- and 18-day lags (*p* < 0.01). Hospitalized COVID-19 patients do not significantly lag EMS encounters.

Appendix Figure A3.1 and Figure A3.2 report how indicators vary in salience across COVID-19 waves. EMS encounters correlates with ICU-occupied beds, in particular in the second wave.

## Discussion

First, there is a significant and positive correlation between the number of new cases identified city-wide and EMS encounters 6 days later (*p* < 0.01), offering city public health officials a new tool for anticipating exposure risks to the frontline personnel, indicating when to heighten infection-control practices to further protect personnel. Through the pandemic, limited ambulance capacity in major cities because of infected and isolating personnel has been a major public health challenge, because of the role EMS services play in supporting individuals experiencing homelessness, addition, behavioral health emergencies, and other complex public health challenges further exacerbated by the pandemic.

Second, EMS caseloads can serve as an early-warning mechanism in urban healthcare systems for COVID-19. There are definitive, positive correlations between EMS encounters and ED COVID-19 patients at a 1-day lag (*p* < 0.01) and with ICU-occupied beds at 7- and 18-day lags (*p* < 0.01). While EMS responses generally lead to transports to emergency departments, the offsets in the significant correlations suggest that Boston EMS may serve a patient sub-population that is getting infected or seeking treatment slightly earlier than the overall population showing up to emergency departments. The EMS data can give hospitals a day of notice of rising COVID-19 patients in emergency departments. Furthermore, the EMS encounters provide reliable 1- to 2-week advance warning of increased numbers of ICU patients. This link enables integrated planning between ambulance services and hospitals [12].

These results highlight the value of using city-level population testing and EMS data together to better understand demand for EMS and risks to frontline personnel, as well as using EMS data alongside hospital data to anticipate acute- and sub-acute-care needs. As cities transition from pandemic to endemic COVID-19, urban healthcare systems are shifting from a response model built on widespread testing that identifies community disease prevalence to a model built around early-warning analytics in order to manage the impact of outbreaks on healthcare system capacity. This shift makes strong early indicators of demand for critical care in city hospitals even more valuable.

This study has several limitations that we make efforts to address. First, with the Box-Jenkins method, we aim to control for autocorrelation inherent in time series data that can produce unmeaningful correlations between variables. Such a retrospective cross-correlation analysis is a promising starting point for future efforts to exploit exogeneity in indicators (e.g., changes in testing access) to identify causal links between indicators and EMS encounters. Second, early in the pandemic, data flows were not fully in place, leading to the possibility of missing data, as seen in Fig. 1.

## Conclusion

This work demonstrates the value of combining population and healthcare-system virus surveillance with EMS data to generate practical insights that can improve frontline personnel safety as well as ambulance system and hospital capacity planning. With clarity on intervals in which community infections, EMS encounters, and acute, and sub-acute-care demand increase, city health officials and partners can more precisely craft public messaging, protection from infection ambulance crews, and plan to surge hospital beds, among other levers in a response.

## Supplementary Information

Below is the link to the electronic supplementary material.

Supplementary file1 (DOCX 7225 KB)
