# Supplementary material for: Policy-Relevant Indicators of Urban Emergency Medical Services COVID-19-Patient Encounters
Source: J Urban Health. 2022 Nov 2;100(1):11–5. doi: 10.1007/s11524-022-00672-0 (PMC9628336; doi:10.1007/s11524-022-00672-0)
Supplement: Supplementary file 1 — Supplementary file1 (DOCX 7225 KB) [file 11524_2022_672_MOESM1_ESM.docx]

**Appendix Table A1.1: Variable source and construction.**

|  | **Source** | **Construction** |
| --- | --- | --- |
|  |  |  |
| **Baseline** |  |  |
| EMS Encounters | Boson Emergency Medical Services | Incidents confirmed to involve a COVID-19 positive patient. |
| **Leading Indicators** |  |  |
| Wastewater Presence Rate | Massachusetts Water Resources Authority | Ratio COVID-19 RNA replicates per mL in Boston wastewater system. |
| New Cases | Boston Public Health Commission | New cases tallied based on one positive test per 90 days per individual. Excludes college students. Is a tally of results of positive lateral flow and polymerase chain reaction tests reported to the state, excluding tests taken and positive and not reported to the state. |
| **Lagging Indicators** |  |  |
| ED COVID-19 Patients | Boston Public Health Commission | Patients making ED visits related to COVID-19. |
| Hospitalized COVID-19 Patients | Boston Public Health Commission | Adult patient new hospitalizations related to COVID-19. |
| ICU Occupied beds | State of Massachusetts | ICU beds occupied (related to COVID-19 or not). |

**Appendix Table A1.2: Variable processing.**

|  | **Processing** |  |  |  |
| --- | --- | --- | --- | --- |
|  | Log-based transform | Weekly adjustment | First difference | KPSS unit root test for stationarity |
| **Baseline** |  |  |  |  |
| EMS Encounters | X |  | X | K = 0.14 with 19 lags for type mu. |
| **Leading Indicators** |  |  |  |  |
| Wastewater Presence Rate |  |  | X | K = 0.14 with 17 lags for type mu. |
| New Cases |  | X | X | K = 0.04 with 18 lags for type mu. |
| **Lagging Indicators** |  |  |  |  |
| ED COVID-19 Patients |  |  | X | K = 0.09 with 17 lags for type mu. |
| Hospitalized COVID-19 Patients | X |  | X | K = 0.30 with 18 lags for type mu. |
| ICU Occupied beds |  |  | X | K = 0.14 with 18 lags for type mu. |
| *Note:* The null hypothesis for the KPSS unit root test for stationarity is that the series is stationary. Significance at 1 percent entails a test statistic of 0.73, significance at 10 percent entails a test statistic of 0.35. | | | | |

**Appendix Table A1.3: Variable modeling.**

|  | **Model and test** |  |
| --- | --- | --- |
|  | Model | Ljung-Box test for autocorrelation |
| **Baseline** |  |  |
| EMS Encounters | ARIMA(0,0,1) | Q = 9.94 at 10 lags and 2 model degrees of freedom with p = 0.27. |
| **Leading Indicators** |  |  |
| Wastewater Presence Rate | ARIMA(1,0,2) | Q = 9.75 at 10 lags and 4 model degrees of freedom with p = 0.13. |
| New Cases | ARIMA(1,0,8) | Q = 6.55 at 13 lags and 10 model degrees of freedom with p = 0.09. |
| **Lagging Indicators** |  |  |
| ED COVID-19 Patients | ARIMA(6,1,1) | Q = 7.38 at 10 lags and 7 model degrees of freedom with p = 0.06. |
| Hospitalized COVID-19 Patients | ARIMA(4,1,7) | Q = 7.61 at 14 lags and 11 model degrees of freedom with p = 0.05. |
| ICU Occupied beds | ARIMA(4,0,3) | Q = 7.42 at 11 lags and 8 degrees of freedom with p = 0.06. |
| *Note:* The null hypothesis for the Ljung-Box test for autocorrelation is that the autocorrelations of a time series are not different from zero. | | |

**Figure A2.1: Cross correlation coefficient of the residuals of EMS COVID-19 patient encounters with daily lag for indicators.**

*Note:* These panels present the cross-correlation coefficient of residuals for ARIMA models for EMS COVID-19 patient encounters and daily lags (leads) for indicators. Significance is noted at the 0.01 level.

**Figure A2.2: Correlation coefficient of EMS COVID-19 patient encounters with daily lag for indicators.**

*Note:* These panels present the coefficients for EMS COVID-19 patient encounters and daily lags for indicators for the pandemic outbreak.

**Figure A3.1: Cross-correlation coefficient of residuals for EMS COVID-19 patient encounters and daily lead for indicators.**

*Note:* These panels present the cross-correlation coefficient of residuals for ARIMA models for EMS COVID-19 patient encounters and daily lags for indicators for the pandemic outbreak and separately for waves one (days 1-185), two (days 186-172), and three (days 473-460). Significance is noted at the 0.01 level.

**Figure A3.2: Cross-correlation coefficient of residuals for EMS COVID-19 patient encounters and daily lag for indicators.**

*Note:* These panels present the cross-correlation coefficient of residuals for ARIMA models for EMS COVID-19 patient encounters and daily lags for indicators for the pandemic outbreak and separately for waves one (days 1-185), two (days 186-172), and three (days 473-460). Significance is noted at the 0.01 level.
